# Estimation of the Motor Threshold for Near-Rectangular Stimuli Using the Hodgkin–Huxley Model

**DOI:** 10.1155/2021/4716161

**Published:** 2021-05-31

**Authors:** Majid Memarian Sorkhabi, Karen Wendt, Marcus T. Wilson, Timothy Denison

**Affiliations:** ^1^MRC Brain Network Dynamics Unit, Nuffield Department of Clinical Neurosciences (NDCN), University of Oxford, Oxford OX1 3TH, UK; ^2^Te Aka Mātuatua-School of Science, University of Waikato, Private Bag 3105, Hamilton 3240, New Zealand; ^3^Department of Engineering Science, University of Oxford, Oxford OX1 3PJ, UK

## Abstract

The motor threshold measurement is a standard in preintervention probing in TMS experiments. We aim to predict the motor threshold for near-rectangular stimuli to efficiently determine the motor threshold size before any experiments take place. Estimating the behavior of large-scale networks requires dynamically accurate and efficient modeling. We utilized a Hodgkin–Huxley (HH) type model to evaluate motor threshold values and computationally validated its function with known true threshold data from 50 participants trials from state-of-the-art published datasets. For monophasic, bidirectional, and unidirectional rectangular stimuli in posterior-anterior or anterior-posterior directions as generated by the cTMS device, computational modeling of the HH model captured the experimentally measured population-averaged motor threshold values at high precision (maximum error ≤ 8%). The convergence of our biophysically based modeling study with experimental data in humans reveals that the effect of the stimulus shape is strongly correlated with the activation kinetics of the voltage-gated ion channels. The proposed method can reliably predict motor threshold size using the conductance-based neuronal models and could therefore be embedded in new generation neurostimulators. Advancements in neural modeling will make it possible to enhance treatment procedures by reducing the number of delivered magnetic stimuli to participants.

## 1. Introduction

Transcranial magnetic stimulation (TMS) is a noninvasive and promising tool to modulate the human cortex and can be used to induce lasting changes in neural activity, with both research and clinical applications [1, 2]. FDA has cleared TMS for the treatment of various neurological and psychiatric diseases and it is under consideration for many other disorders. In TMS devices, strong, brief current stimuli driven through a wire-wound coil induce an electric field to modulate nerve cells. Despite the success of the TMS procedure, there are several critical limitations associated with the magnetic pulse shape parameters. Conventional neurostimulators most commonly employ LC resonant circuits, resulting in a constant waveform for the magnetic pulses, depending on the predetermined hardware [3, 4].

More adjustable control of the magnetic field waveform can potentially enable new research and clinical applications that are not realizable with conventional TMS tools, such as changing the waveform of the magnetic pulse [4]. Addressing this need, Peterchev et al. have introduced a controllable-TMS (cTMS) machine to produce flexible near-rectangular stimulus shapes with three different architectural variations [5]. Utilizing the concept of pulse width modulation (PWM), Sorkhabi et al. can synthesize arbitrary waveforms [6, 7]. These Instruments open a new parameter space for magnetic stimulation by producing and manipulating a magnetic pulse whose pulse shape has been impressively managed. With the control of PW and pulse direction, neural populations could be selectively targeted [8].

The motor threshold (MT) is one of the standard quantifiable measures of corticospinal excitability in TMS research and is used to determine the TMS intensity. The MT is usually defined as the lowest stimulation intensity able to produce motor-evoked potentials (MEP) of size 0.05 mV with a 50% probability in the abductor pollicis brevis [9]. The MT determination might be measured in the relaxed muscle (resting MT or RMT) or during active muscular contraction (AMT). Estimating the MT can beneficially improve the safety implications of preintervention probing in the magnetic stimulation tests by minimizing the number of delivered pulses to individuals [10]. This modeling also allows the optimization of stimulation waveforms in terms of the required minimum hardware/energy to generate them. As a result, the possibility of optimizing simulation-based tools will be enabled.

## 2. Techniques and Methods

### 2.1. cTMS Circuit

**Figure 1:**
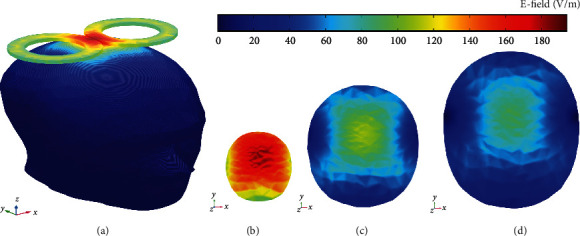
FEM simulation of E-field norm distributions for different distances from the coil surface. (a) Placing figure-8 coil on the skull and the generated E-field. (b) E-filed 5 mm under the coil surface. (c) E-filed 10 mm under the coil surface. (d) E-field 15 mm under the coil surface. For this model, a 70 mm figure-8 coil is connected to 1 kV. The E-field norm means the amplitude of the electric field ($\sqrt{E_{x}^{2}+E_{y}^{2}+E_{z}^{2}}$).

In the current work, we simulate the cTMS circuit to generate different near-rectangular pulses, directions, and intensities. Four insulated-gate bipolar transistors (IGBTs) switches and freewheeling diodes, which form the two half-bridges structure, were used to connect the treatment coil to the DC pulse capacitors. Two isolated DC supplies (*V*_DC1_, −*V*_DC2_) have been used to charge the DC pulse capacitors and the output stimulus can have four different voltage levels (*V*_coil_ = {*V*_DC1_, −*V*_DC2_, *V*_DC1_ − *V*_DC2_, 0}). The cTMS circuit structure with the parameters presented in [11] was simulated in the MATLAB Simulink environment (Powergui blockset, R2020a). The circuit model of this device and its typical parameters are shown in Figure 1S, supplementary data. The simulated results were used in the Hodgkin–Huxley (HH) model to estimate the MT.

### 2.2. Overview of the HH Model

The HH equations have been widely used in the modeling of several neuromodulation modalities and neuronal behavior [12–14]. In this study, for the computational modeling of cortical neurons, a Hodgkin–Huxley type model is selected, including four conductances (the voltage-dependent sodium, potassium, slow potassium, and resting (leak) membrane conductance) and the TMS-induced current density. This model has biophysically meaningful and measurable parameters and allows researchers to investigate the interplay between an external stimulus current and an ionic current. To establish this conductance-based neuronal model, we review experimental data from various preparations and obtain a computational model that captures the fundamental aspect of the intrinsic characteristics utilizing a minimal number of equations. The HH model is more realistic than the RC integrated-fire model and can be fit for physiological measurements. The main equations and parameters are derived from [15–17] and the required parameters to apply the magnetic stimulation are added according to the following equations:

(1) $$\begin{array}{c} C_{m}\frac{\text{d}V_{m}}{\text{d}t}=\;-\;J_{\text{leak}}\;-J_{\text{Na}}\;-J_{\text{K}}\;-J_{\text{M}}\;-J_{\text{stim}}, \end{array}$$

(2) $$\begin{array}{c} J_{\text{leak}}=\;g_{\text{leak}}\left(V_{m}\;-\;E_{\text{leak}}\right), \end{array}$$

(3) $$\begin{array}{c} J_{\text{Na}}=g_{\text{Na}}\left(V_{m}-E_{\text{Na}}\right), \end{array}$$

(4) $$\begin{array}{c} J_{\text{K}}=\;g_{\text{K}}\left(V_{m}-E_{\text{K}}\right), \end{array}$$

(5) $$\begin{array}{c} J_{\text{M}}=g_{\text{M}}\;\left(V_{m}-E_{\text{K}}\right), \end{array}$$

(6) $$\begin{array}{c} g_{\text{Na}}=\;{\bar{g}}_{\text{Na}}\;m^{3}h, \end{array}$$

(7) $$\begin{array}{c} g_{\text{K}}={\bar{g}}_{\text{K}}n^{4}, \end{array}$$

(8) $$\begin{array}{c} g_{\text{M}}={\bar{g}}_{\text{M}}p, \end{array}$$

(9) $$\begin{array}{c} \frac{\text{d}m}{\text{d}t}=\alpha_{m}\left(V_{m}\right)\left(1-m\right)-\;\beta_{m}\left(V_{m}\right)m, \end{array}$$

(10) $$\begin{array}{c} \frac{\text{d}h}{\text{d}t}=\;\alpha_{h}\left(V_{m}\right)\left(1-h\right)-\;\beta_{h}\left(V_{m}\right)h, \end{array}$$

(11) $$\begin{array}{c} \frac{\text{d}n}{\text{d}t}=\;\alpha_{n}\left(V_{m}\right)\left(1-n\right)-\;\beta_{n}\left(V_{m}\right)n, \end{array}$$

(12) $$\begin{array}{c} \frac{\text{d}p}{\text{d}t}=\frac{\left(p_{\infty }\left(V_{m}\right)-p\right)}{\tau_{p}\left(V_{m}\right)}. \end{array}$$

The *C*_*m*_ term represents the membrane lipid bilayer capacitance. The symbols *J*_Na_, *J*_K_, *J*_M_ and *J*_leak_ denote the current densities of sodium, potassium, slow potassium (delayed rectifier and slow nonactivating current), and other leakage ions, respectively, whereas *J*_stim_ is the TMS-induced current density by the cTMS stimulus (units: *µ*A/cm^2^). ${\bar{g}}_{\text{Na}}$, ${\bar{g}}_{\text{K}}$ and ${\bar{g}}_{\text{M}}$ are the maximal values of the conductances for the voltage-dependent sodium, potassium, and slow potassium currents per unit area. The dimensionless gating variables *n*, *m*, and *h* are associated with potassium channel activation, sodium channel activation, and sodium channel inactivation, respectively, and are bounded between 0 and 1. The transition rates between open and closed states of the ion channels (*α*_*i*_ and *β*_*i*_, *i* = *m*, *n*, *h*) are positive coefficients that nonlinearly depend on the membrane potential *V*_*m*_, as determined below:

(13) $$\begin{array}{c} \alpha_{m}\left(V_{m}\right)=\;\frac{-0.32\;\left(V_{m}-V_{T}-13\right)}{e^{-\left(V_{m}-V_{T}-13\right)/4\;}-1},\\ \beta_{m}\left(V_{m}\right)=\frac{0.28\left(V_{m}-V_{T}-40\right)}{e^{\left(V_{m}-V_{T}-40\right)/5}-1},\\ \alpha_{h}\left(V_{m}\right)=0.128\left(e^{-\left(V_{m}-V_{T}-17\right)/18}\right),\\ \beta_{h}\left(V_{m}\right)=\;\frac{4}{1+e^{-\left(V_{m}-V_{T}-40\right)/5}},\\ \alpha_{n}\left(V_{m}\right)=\frac{-0.032\left(V_{m}-V_{T}-15\right)}{e^{-\left(V_{m}-V_{T}-15\right)/5}-1},\\ \beta_{n}\left(V_{m}\right)=0.5\;e^{-\left(V_{m}-V_{T}-10\right)/40},\\ p_{\infty }\left(V_{m}\right)=\frac{1}{1+e^{-\;\left(V_{m}+35\right)/10}},\\ \tau_{p}\left(V_{m}\right)=\;\frac{\tau_{\max}}{3.3\;e^{\left(V_{m}+35\right)/20}+e^{-\;\left(V_{m}+35\right)/20}}, \end{array}$$

where *p*_*∞*_ and *τ*_*p*_ are the steady-state activation and time constant for the slow potassium current, respectively. As seen in equations (3)–(8), the conductances of the sodium and the potassium channels (*g*_Na_, *g*_K_ and *g*_M_) are a nonlinear function of time and the membrane voltage *V*_*m*_, while this parameter is constant for the leak channels. The details of the variables and their values involved in these equations are provided in Table 1.

### 2.3. Solver

The equations of the single-compartment Hodgkin–Huxley type model were solved in MATLAB with Euler's numerical solution method (timestep: d*t* = 1 *μ*s, number of iterations: 20000). To find a relationship between the treatment coil voltage (stimulation intensity) and the corresponding induced current in the cortex, the finite-element method (FEM) and 3D MRI-derived head model are used to compute the induced Electric-field (E-field). The head tissue of a healthy subject is assumed to be homogeneous with an electrical conductivity of 0.27 S/m [18]. E-field distributions were calculated with COMSOL Multiphysics 5.3 for a Magstim 70 mm figure-of-8 coil (P/N 9925, 3190). The specifications of the figure-8 coil are derived from [19], which has 9 windings in each lobe with inner and outer diameters of 5.2 and 8.8 cm, respectively. The final mesh structure of the head comprised 120,000 nodes and 2.4 million tetrahedral elements.  Figure 1 shows E-field norm distributions on planes 5, 10, and 15 mm beneath the coil, where the coil-to-scalp distance is 2 mm and the peak coil voltage is 1 kV. Given that the target hand motor area for finding the MT is generally assumed to be 15 mm below the scalp, it can be concluded from the FEM results that for every thousand volts of a figure-8 coil voltage, an electric field of 100 V/m is induced in the motor hand knob [20]. Therefore, the induced current to the target area will be 27 A/m^2^ per kV (*J*_stim_ in equation 1).

The results of human tests that have previously shown the relationship between the MT and the conventional monophasic and biphasic stimuli [21–23] have been used to validate the HH equations and ratios obtained by the FEM modeling. It was found that the FEM method and reported experimental results are consistent with an accuracy of ±4.6%. The approximation of the neuron's membrane as a low-pass RC filter and experimentally measuring the membrane potential changes with a pickup coil also shows similar results. For more details of this measurement, see [6].

We set the transmembrane current density driving the HH model to this value. While transmembrane current densities are in practice not equal to the induced current densities, being dependent on cellular and brain geometry and conductivity variations, we expect them to be strongly related. We identify motor threshold as being the pulse-amplitude required to produce an action potential in the HH model for a given pulse shape. Resting motor thresholds are consistently higher than AMTs, usually by about 10% of the stimulator output [24]. There is also a positive correlation between participant's resting and active MTs [25, 26]. Therefore, by finding one of these two, the other value can be estimated.

### 2.4. Database

To validate the estimated MT, we used the results of human experiments reported by [5, 8, 27–29], in which 50 individuals were exposed to the different magnetic pulses of the cTMS neurostimulator. The data was collected from three types of cTMS waveforms, monophasic, unidirectional, and bidirectional in posterior-anterior (PA) or anterior-posterior (AP) directions. In those studies, the relationship between the pulse width and the effectiveness of a magnetic pulse (i.e., the strength-duration relationship) is specifically considered. A summary table indicating the number, population age, gender, and health conditions of subjects are available in Table 1S, supplementary data.

**Figure 2:**
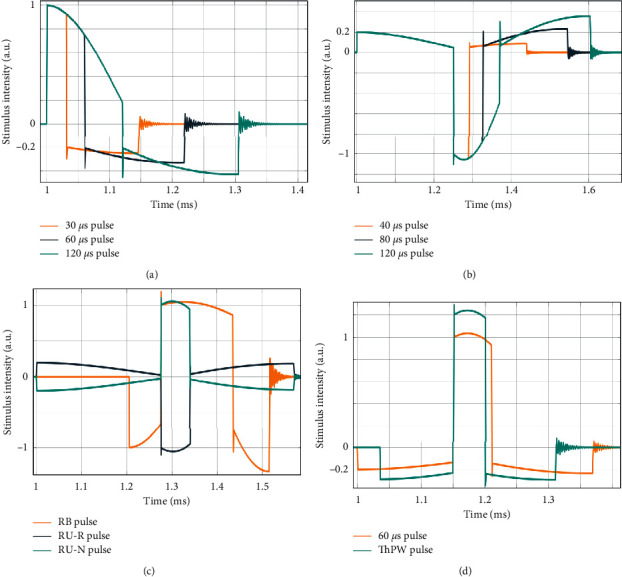
cTMS device pulses as monophasic, unidirectional, and bidirectional waveforms, normalized to unity amplitude which have been tested in different human experiments. (a) Monophasic pulses with positive pulse widths of 30, 60, and 120 *μ*s in [5, 27] with initial PA direction. (b) Unidirectional pulses for negative pulse widths of 40, 80, and 120 *μ*s in [8] with initial AP direction. (c) Three different unidirectional and bidirectional waveforms in [28]; RB : Rectangular bidirectional (*m* = 1). RU-N : Rectangular unidirectional with initial AP direction (*m* = 0.2). RU-R : Rectangular unidirectional with initial PA direction. (d) Unidirectional pulses for positive pulse widths of 60 *μ*s and the minimum threshold pulse width (ThPW) in [29] with initial AP direction (*m* = 0.2). The ratio of the peak of the negative phase to the positive phase is called the m ratio. Generally, the stimulus intensity is proportional to the E-field, (a derivative of the B-field), i.e., the current density in the brain and input to the HH model.

**Figure 3:**
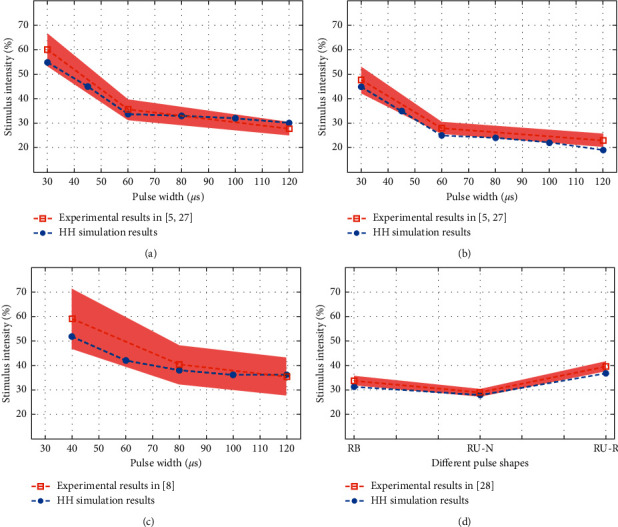
The effect of different pulse shapes on the MT (mean ± standard deviation). Strength-duration curves for (a) RMT measurement results and (b) AMT measurement results for monophasic pulses in [5, 27] (*n* = 10), which are compared to the simulation results of the HH model, (c) RMT measurement results for unidirectional pulses in [8] (*n* = 15). (d) Resting motor thresholds for RB, RU-N, and RU-R pulses in [28] (*n* = 13), in comparison with the modeling results. The stimulus intensity reflects the MT as a percentage of the maximum stimulator output. The maximum voltage of the cTMS device is 2800 V which is equivalent to 100% stimulus intensity. The filled red area shows the standard deviation of the mean, according to the experimental results. All estimated MT values are inside or very close to the area of the standard deviation.

In [5, 27], the effect of three monophasic stimuli (pulse width, i.e., positive phase duration, of 30, 60, 120 *μ*s) on the RMT and the AMT was examined. These pulses are shown in Figure 2(a) and the measured RMT and AMT are displayed in Figures 3(a) and 3(b), respectively. As reported in those studies, by increasing the PW, the intensity of the stimulus required to see the defined MEP decreases. Also, the RMT is approximately 10% higher than the AMT.

In [8], the effect of three unidirectional stimuli (negative pulse width of 40, 80, 120 *μ*s) on the RMT and the plastic aftereffects were studied. These pulses are shown in Figure 2(b) and the measured RMTs are displayed in Figure 3(c).

Goetz et al. quantified changes in the corticospinal excitability produced by three near-rectangular-shaped cTMS stimuli, one bidirectional and two unidirectional in opposite directions (named RB : Rectangular bidirectional. RU-N : Rectangular unidirectional with initially AP. RU-R : Rectangular unidirectional with initially PA) [28]. These pulses are shown in Figure 2(c) and the measured RMTs are displayed in Figure 3(d).

In [29], the plasticity effect of unidirectional cTMS pulses in the paired-pulse protocol was investigated. Two types of MT were measured in that research.

  #1RMT : resting motor threshold for a positive pulse width of 60 *μ*s and *m* = 0.2.
  #2RMT : The pulse magnitude was then set to 120% of #1RMT, which the authors called the “test intensity.” They then measured a threshold pulse width (ThPW) that elicits resting MEPs with a 50% probability. According to the results of this study, the average #1RMT is 32% of maximum stimulator output (MSO) and the ThPW of #2RMT is 47.3 *μ*s, as shown in Figure 4.

Although we call the pulses by their higher pulse widths in terms of absolute amplitude in the cTMS device, such as 40 *μ*s or 80 *μ*s pulses, in all calculations, the compensatory phases are also included in the waveform and the MT estimation. Selecting the width of the major phase is arbitrary and the opposite phase is selected to reach the almost zero current at the end of each pulse. *M* ratio has a direct effect on the choice of positive and negative pulse widths to reach this goal. Also, due to some limitations of the cTMS equipment in charging and discharging high voltage DC capacitors, for repetitive or paired pulses protocols, balanced pulses must be defined.

The shape of the cTMS pulses was selected based on the experiments defined in our database, and their purpose was to investigate the effect of different pulse shapes on the neural behavior, both monophasic and biphasic pulses. Starting or ending with a compensatory phase was done by researchers in our selected databases, and we have no role in defining pulses. We simulated their selected waveforms and estimated the MT.

## 3. Results

**Figure 4:**
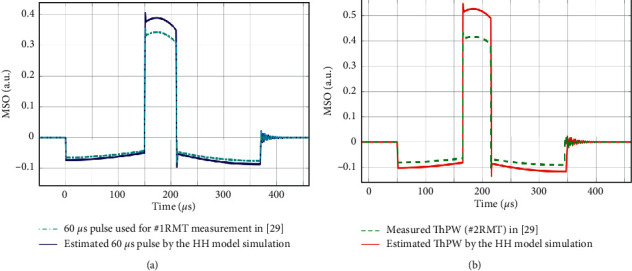
Results of human experiments [29] and HH modeling results of (a) the threshold intensity for a 60 *μ*s unidirectional pulse, according to the #1RMT protocol and (b) the minimum threshold pulse width (ThPW), according to the #2RMT protocol. The amplitude of each estimated pulse indicates the minimum intensity required to stimulate the neuron and to generate an action potential in the HH model (continuous lines). The amplitude of each tested pulse indicates the minimum intensity required to elicit minimal MEPs in the experimental trials (dashed lines).

The estimated MT values and the experimental values in the defined database can be seen in Figures 3 and 4. The maximum absolute error between the average of the RMT and the AMT for monophasic stimuli (assumed as the true motor threshold) [5, 27] and the MT estimates made with the HH model was 5% at 30 *μ*s and 4% at 120 *μ*s PW, respectively. The maximum absolute error between the average of the RMT for the three unidirectional stimuli [8] and the estimated MTs was 8% at 40 *μ*s PW. The error between the average of the RMT for one bidirectional and two unidirectional (in opposite directions) stimuli [28] was 3% for the RU-R pulse.

According to the #1RMT protocol in [29], the 60 *μ*s stimulus intensity is estimated to be 39.2% of MSO to generate an action potential in the HH model. According to the #2RMT protocol, the ThPW is estimated to be 50 *μ*s to evoke a response. Therefore, the maximum absolute error between the #1RMT and #2RMT protocols and the MT estimates obtained with the HH model was 7.2% and 5.7%, respectively. The measurement and estimation results are shown in Figure 4.

## 4. Discussion

In this research, the novel method to predict the RMT and the AMT was introduced. The results of human experiments presented in five previously published studies, which used the cTMS device for their trials, were collected as a database. Based on the outcomes of the computational modeling and the comparison with the database results, it can be concluded that the proposed method can estimate the values of the MT for all three types of monophasic, unidirectional, and bidirectional pulses with different directions and widths.

In a similar attempt, Julkunen applied the cumulative distribution function (CDF) with Monte Carlo simulations to determine an MT estimate [9]. Although the precision of that method is higher than in this study (error ≤ 0.9%), its algorithm can only be used for conventional pulses [30], and it is not possible to predict the MT value by manipulating the stimulus character (such as the waveform, frequency, and directionality).

Recent computational and experimental studies highlight the complexity in the MT estimation with TMS. Wu et al. implemented the cable theory which was adjusted in the detailed compartmental neural cell models [31]. Aberrra et al. utilized the FEM with morphologically realistic models of nerve cells to estimate cortical response to the different magnetic stimuli and quantified the activation threshold for layers 1–6 [32]. Wilson et al. used a neural field approach with a nonlinear model of motoneuron firing to model motor-evoked potentials as a function of amplitude, including identification of motor thresholds [33]. However, despite comprehensive approaches, the increasing sophistication of these computational models dramatically reduces their computational efficiency [34]. In contrast, this present work demonstrates that a straightforward calculation based on the well-established HH model and finite-element modeling of electric fields in a simple geometry is sufficient to reproduce experimental results for motor thresholds. However, more complicated approaches may well be required for modeling effects above the threshold, for example, the size of motor-evoked potentials.

Finally, we emphasize that the model has reproduced experimental results without tuning parameters. We have taken a standard implementation of the HH model and made a physical estimate of the induced current density *J*_stim_ for a given applied voltage to the coil with no fitting of parameters.

### 4.1. Limitation

Evidently, the effects of magnetic pulses on neural tissues are complex and are a function of multiple variables, including a contribution from the brain (gyral) geometry [35], the cortex position [36], the size of the somatic compartment, and the input resistance [37]. Modeling these parameters requires the implementation of complex equations and possibly FEM for geometric modeling of the gyral crown. Thus, the proposed method cannot predict RMT or AMT for individuals but can predict average thresholds for a population of people. It may be possible to individualize the approach by changing the mapping between peak coil voltage and induced current density *J*_stim_ for different people based on a standardized pulse shape response.

In this study, we focused on the activation kinetics of the voltage-gated ion channels. We have assumed that the input current density to the HH neuron is directly related to the induced electric field due to the TMS pulse. Despite the many simplifications, all of our modeling results have clearly correlated with previous works. Using the assumption that ion channel modeling can predict the overall strength-duration trend with an acceptable error range, it may be possible to estimate the intensity for the stimulus by embedding the differential equations in the hardware.

Additionally, several ions are assumed to be involved in the activation kinetics of the ion channels stimulated by near-rectangular pulses, but a possible connection between the pharmacological impact on motoneuron firing has not been determined yet. Considering that the MEP creation was affected by the administration of neuroactive drugs which are related to neurotransmitters [38], further pharmacological research is needed to describe the effect of magnetic pulse shapes and the MEP.

### 4.2. Innovation

For the first time, the suggested approach has been shown to estimate the MT for cTMS stimuli. The MT assessment allows investigators to refine the strength-duration relationship for controllable stimuli. Finding the optimal pulse with the minimum energy required to stimulate the neuron can steer to the plan of a novel magnetic pulse generator equipment with minimal hardware and size. This issue can be useful in home-TMS equipment, especially now that there is an urgent need for more accessible at-home neurotherapeutics throughout this COVID-19 social isolation period [39]. The prediction of the MT can also minimize the number of experiments required to identify the MT for new magnetic stimuli.

### 4.3. Sensitivity Analysis of Hodgkin–Huxley Model to Parametric Variations

**Figure 5:**
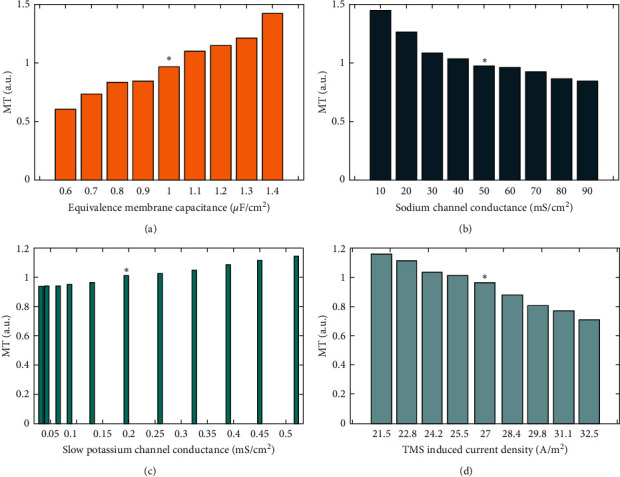
Sensitivity analysis results for the parameters of the HH equations. The MT changes, depending on (a) membrane capacitance changes from 0.6 to 1.4 *μ*F/cm^2^, (b) sodium channel conductance changes from 10 to 90 mS/cm^2^, (c) slow potassium channel conductance changes from 0.0325 to 0.52 mS/cm^2^, (d) TMS-induced current density changes from 21.5 to 32.5 (A/m^2^). MT sizes are normalized with the experimentally measured MT for the RU-N stimuli. The ∗ marker represents the standard values utilized in this study to estimate the MT.

Due to the regulation of the excitability of nerve cells by several ion channels and also membrane capacitance, a critical inquiry for the model accuracy is to find the sensitivity of neural behavior to a variation in HH parameters: which value is most important for the MT estimation? To simplify sensitivity analysis, we focus on RU-N pulse and 7 parameters, which are assumed to change independently and all remaining parameters keep unchanged and ignore changes in equilibrium potentials. Analyzed parameters include equivalence membrane capacitance (*C*_*m*_), sodium channel conductance (${\bar{g}}_{\text{Na}}$), potassium channel conductance (${\bar{g}}_{\text{K}}$), slow potassium channel conductance (${\bar{g}}_{\text{M}}$), leak channel conductance (*g*_leak_), time constant for the slow potassium channel (*τ*_max_), and TMS-induced current density (*J*_stim_). The calculated results for changing the estimated MT value for this sensitivity analysis are shown in Figure 5.

The range of changes in the parameters has been selected according to the different values measured in different references, biological variation in the parameters [15], and the possibility of observing the change in the estimated value of the MT. The electrical conductivity variation is assumed to be ±20%, based on the in vitro measurements presented in [40].

According to the computational sensitivity results, it can be seen that the highest sensitivities are related to the values of membrane capacitance and sodium channel parameters. The sensitivity of the MT to the values of potassium and TMS-induced current density is less than the previous parameters. Also, the HH equations are almost insensitive to fluctuations in leak channel conductance, potassium channel conductance, and time constant for the potassium channel, which are not shown in Figure 5. Mathematical analysis of the neuronal model, instead of computational analysis, can calculate more accurate results of the sensitivity of the proposed method [15, 41].

## 5. Conclusion

cTMS equipment has opened up novel opportunities to investigate the effect of magnetic pulse shape on the human nervous system. In this study, we introduce a novel method for determining MTs for cTMS device pulses based on the HH model, as mathematical descriptions of neuronal behavior that is exposed to the magnetic stimulation. The HH model was selected as an interconnection of a biochemical network with mathematical neuroscience. The results of this computational study highlighted a strong contribution to the activation kinetics of the voltage-gated ion channels for the motor threshold induced by TMS. Validation with previously published experimental threshold data confirms that the proposed method captures the measured population-averaged MT value at high precision, without the need to perform finite-element modeling in realistic head geometries or consideration of large networks or populations of cells. The proposed prediction method will allow a rapid estimation of the motor threshold to aid decision-making by researchers and clinicians to determine the motor threshold and improve the safety implications of preintervention probing. Furthermore, the proposed mathematical framework might be a promising tool to explore efficient magnetic pulses to design a home-TMS machine.

## Figures and Tables

**Table 1:** Parameters used for the HH model.

Variables	Definitions	Unit	Values
*C*_*m*_	Equivalence membrane capacitance	*μ*F/cm^2^	1
*g*_leak_	Leak channel conductance	mS/cm^2^	0.016
${\bar{g}}_{\text{Na}}$	Max. value of the sodium channel conductance	mS/cm^2^	50
${\bar{g}}_{\text{K}}$	Max. value of the potassium channel conductance	mS/cm^2^	4.8
${\bar{g}}_{\text{M}}$	Max. value of the slow potassium channel conductance	mS/cm^2^	0.13
*E*_leak_	Nernst potentials of leakage ions	mV	−70.3
*E*_Na_	Nernst potentials of sodium ions	mV	50
*E*_K_	Nernst potentials of potassium ions	mV	−90
*V*_T_	Voltage to adjust the spike threshold	mV	−61.5
*τ*_max_	Max. time constant for the slow potassium channel	ms	1123.5

## Data Availability

No data were used in the study.

## Supplementary Materials

Supplementary Materials Figure 1S: diagram of key circuit elements of the controllable-TMS device. Table 1S: baseline demographic of the database.
