# Supplementary material for: Estimation of the Motor Threshold for Near-Rectangular Stimuli Using the Hodgkin–Huxley Model
Source: Comput Intell Neurosci. 2021 May 31;2021:4716161. doi: 10.1155/2021/4716161 (PMC8184325; doi:10.1155/2021/4716161)
Supplement: Supplementary Materials — Figure 1S: diagram of key circuit elements of the controllable-TMS device. Table 1S: baseline demographic of the database. [file 4716161.f1.docx]

**+**

C_1_

C_2_

Coil

V_DC1_

V_DC2_

+

_

+

_

Q_1_

Q_2_

Q_3_

Q_4_

D_1_

D_2_

D_3_

D_4_

Figure 1S Diagram of key circuit elements of the controllable-TMS device, where typically C_1_= 430 µF, C_2_= 2 mF, V_DC1(max.)_= 2.6 kV, V_DC2(max.)_= 1kV, Q_1_-Q_4_ are the Insulated Gate Bipolar Transistors (IGBTs) and D_1_-D_4_ are the freewheeling diodes.

Table 1s Baseline demographic of the database

| Reference | Number of subjects | Gender (Male-Female) | Age (years± SD) | Condition |
| --- | --- | --- | --- | --- |
| [5][27] | 10 | 5-5 | 29 ± 5 | Right-handed healthy subjects |
| [28] | 15 | 5-15 | 25.2±3.7 | Right-handed healthy subjects |
| [29] | 21 | 7-14 | age range: 18–48 years | Right-handed healthy subjects |
| [30] | 12 | 6-6 | 24.4±3.6 | Right-handed healthy subjects |
